# Management of Intractable Pain in Patients With Implanted Spinal Cord Stimulation Devices During the COVID-19 Pandemic Using a Remote and Wireless Programming System

**DOI:** 10.3389/fnins.2020.594696

**Published:** 2020-12-08

**Authors:** Yang Lu, Duo Xie, Xiaolei Zhang, Sheng Dong, Huifang Zhang, Beibei Yu, Guihuai Wang, James Jin Wang, Luming Li

**Affiliations:** ^1^Department of Neurosurgery, Beijing Tsinghua Changgung Hospital, School of Clinical Medicine, Tsinghua University, Beijing, China; ^2^National Engineering Laboratory for Neuromodulation, School of Aerospace Engineering, Tsinghua University, Beijing, China; ^3^Precision Medicine and Healthcare Research Center, Tsinghua-Berkeley Shenzhen Institute, Tsinghua University, Shenzhen, China; ^4^IDG/McGovern Institute for Brain Research at Tsinghua University, Beijing, China; ^5^Institute of Epilepsy, Beijing Institute for Brain Disorders, Beijing, China

**Keywords:** chronic intractable pain, spinal cord stimulation, remote programming, telemedicine, COVID-19

## Abstract

As COVID-19 rampages throughout the world and has a major impact on the healthcare system, non-emergency medical procedures have nearly come to a halt due to appropriate resource reallocation. However, pain never stops, particularly for patients with chronic intractable pain and implanted spinal cord stimulation (SCS) devices. The isolation required to fight this pandemic makes it impossible for such patients to adjust the parameters or configuration of the device on site. Although telemedicine has shown a great effect in many healthcare scenarios, there have been fewer applications of such technology focusing on the interaction with implanted devices. Here, we introduce the first remote and wireless programming system that enables healthcare providers to perform video-based real-time programming and palliative medicine for pain patients with a SCS implant. During the COVID-19 pandemic from January 23, 2020, the date of lockdown of Wuhan, to April 30, 2020, 34 sessions of remote programming were conducted with 16 patients. Thirteen of the 16 patients required programming for parameter optimization. Improvement was achieved with programming adjustment in 12 of 13 (92.3%) cases. Eleven of the 16 (68.8%) patients reported that the system was user-friendly and met their needs. Five patients complained of an unstable connection resulting from the low network speed initially, and three of these patients solved this problem. In summary, we demonstrated that a remote wireless programming system can deliver safe and effective programming operations of implantable SCS device, thereby providing palliative care of value to the most vulnerable chronic pain patients during a pandemic.

**Clinical Trial Registration:**

www.clinicaltrials.gov, identifier NCT 03858790.

## Introduction

The world has changed rapidly due to the disastrous Coronavirus Disease 2019 (COVID-19) pandemic. By the end of July 2020, there were over 19 million confirmed cases of infection and 712,000 deaths due to COVID-19 worldwide. Many countries have issued strict isolation measures or have at least advocated “social distancing” in people’s daily lives. Governments and medical providers have had to reallocate labor and material resources to deal with this abrupt emergency. Thus, elective operations/therapies and non-emergency medical procedures have nearly all come to a halt in most medical centers. Patients are also avoiding visiting medical facilities that may be considered a potential source of infection (Keesara et al., 2020). Consequently, some medical needs could be delayed and not met. For patients with demands that cannot be delayed and are impossible to fulfill with the medical resources on site because of isolation protocols or resource limitations, telemedicine has offered an alternative solution (Keesara et al., 2020).

Telemedicine has gradually been adopted by patients over the last decade (Adamse et al., 2018). During the current COVID-19 pandemic, the demand for treatment at a distance from healthcare providers surged essentially overnight (Calton et al., 2020; Humphreys et al., 2020). These changed conditions have boosted reform of the healthcare system. In response, the Chinese government launched a series of policies to support the development of telemedicine and “cloud hospitals.” In March 2020, the United States government announced that telemedicine services would be reimbursed by the Medicare and Drug Enforcement Administration, who allowed the prescription of controlled medicine via telemedicine (ATA, 2020).

In most pain management settings, telemedicine has been used in the form of video consultation, which mainly focuses on complaints that can be orally solved, such as palliative care and self-management skills. Some reviews have identified small to moderate reductions in pain, disability, and psychological symptoms in intervention groups applying telemedicine when compared with the control (including standard pain care or waitlist control) (Buhrman et al., 2016; Mariano et al., 2019). However, no therapeutic difference was found when compared to the active control (such as on-site therapies) (Mariano et al., 2019). Remotely delivered physical exercise interventions have also been proposed to reduce pain in patients as a substitute for the usual care (Adamse et al., 2018). However, for addressing more specific demands in a complex situation such as for a patient with chronic intractable pain who has a spinal cord stimulation (SCS) implant, video-based consultation alone is not sufficient. These patients usually require several programming sessions (mostly over 3 months) to achieve a stable therapeutic effect after implantation. When people with chronic pain are deprived of appropriate assessment and treatment, their condition can worsen significantly. Lead migration, scar-related impedance change, and habituation to the therapy require further programming and regular adjustments (North et al., 2005; Kumar et al., 2008). Considering that patients with chronic pain usually suffer from depression and anxiety, further negative emotions brought on by COVID-19 could exacerbate the pain (Eccleston et al., 2020). These unmet demands require multi-dimensional interactions and teleprogramming of the stimulator by distance.

Here, we introduce the first time remote and wireless programming system for a SCS implant, which enables chronic pain patients to receive timely adjustment of parameters and configurations with only a smartphone and access to a 4G/5G/WiFi network at home.

## Materials and Methods

### Participants

The patients in this study were recruited for a multi-center clinical trial (Lu et al., 2020). Patients with chronic intractable pain were recruited at our center from January 2019 to December 2019. Patients were screened from electronic medical records and then approached to complete the verification of the inclusion/exclusion criteria and provided consent. The inclusion criteria were the following: (1) chronic intractable pain that has lasted for at least 3 months and is refractory to the conservative therapies, including oral medications, nerve block, epidural corticosteroids, physical and psychological rehabilitation therapy, and chiropractic care; (2) aged over 18; (3) good compliance and ability to complete post-operative follow-up; and (4) understanding of the method and willingness to sign the informed consent. The exclusion criteria were the following: (1) pregnancy, breast feeding, plan to be pregnant or unwilling to use contraceptive methods; (2) bleeding complications or coagulation disorders; (3) severe mental or cognitive disorders, leading to inability to cooperate during surgery and post-operative programming; (4) life expectancy of less than one-year; (5) need for therapy or examination that could not be provided with an implanted pulse generator (IPG), such as magnetic resonance imaging and thermo-therapy; and (6) other inappropriate situations that were determined by the investigators.

All patients were implanted with IPG successfully, including 10 males and six females, with an average age of 60.1 ± 9.9 years (range 39–74 years old). None of the patients had a device infection except for one patient who complained of red skin at the IPG incision site but had no fever or pain. Two female patients had mild discomfort at the IPG site, but no further surgical treatment was required. There were three cases of spinal cord injury, two cases of peripheral nerve injury, two cases of brachial plexus avulsion, two cases of failed back surgery syndrome, and one case each of complex regional pain syndrome, neuropathic pain, amputated limb pain, phantom limb pain, failed cervical surgery syndrome, syringomyelia, and lower limb ischemic pain in this study.

### Intervention

In accordance with standard clinical practice (Kapural et al., 2015), patients first underwent a screening phase of SCS (with a percutaneous lead or paddle lead) lasting up to 10–14 days with an external stimulator to determine the short-term response. Patients that experienced 50% or greater pain reduction based on the visual analog scale from baseline were then eligible to proceed to permanent implantation.

Stimulation parameters were adjusted to optimally overlap paresthesia with the region of the pain area when the patients left the hospital (mean ± SD of the minimum and maximum programmed parameters: frequency, 70.2 ± 30.0 Hz; amplitude, 4.6 ± 2.8 mA; and pulse width, 310 ± 148 μs). Subsequently, intraoperative paresthesia testing and associated device programming were performed in the hospital, as needed based on patient feedback in standard clinic visits. Oral analgesics were stabilized from 28 days before enrollment until activation of the implanted SCS system, in addition to perioperative analgesics. Adjustments were then allowed under the guidance of the attending physician as medically necessary.

### Introduction of the Remote Programming System

Based on our previously developed telemedicine system (Chen et al., 2014, 2015c), the SCS remote programming system, called the PINS remote programming system, was developed in 2019. The PINS remote programming system can provide multi-dimensional interaction between healthcare providers and chronic pain patients through a computer with PINS remote programming software installed on the physician side, a smartphone with the PINS remote programming application and patient’s external programmer on the IPG patient side, and a 4G/5G/WiFi network with an upload speed of no less than 1 MB.

**FIGURE 1:**
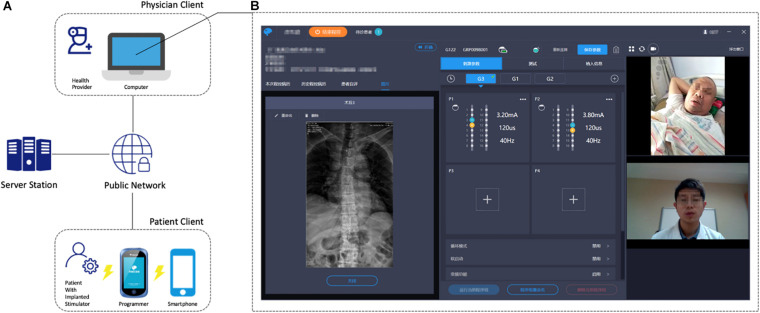
**(A)** General architecture of the remote programming system. **(B)** Physician client illustration with the real-time video consultation (right), parameter and configuration adjustment (middle), and the patient’s medical record (left). The patient sent a WebRTC request to the signaling server, which is subsequently relayed to the physician client. If an online physician replies to the WebRTC request, a P2P connection will be established, and video stream is facilitated between the two clients.

Before the connection, the smartphone and the external programmer-activated IPG were paired. Then, according to the patients’ requests or previously stated reservation, the healthcare provider logged into the programming system and established the connection with the patient’s smartphone after activation of the IPG with patient’s external programmer. The smartphone could receive bidirectional, high-definition, and real-time video signals and programming instructions using the public network. The connections between the smartphone and IPG are generated via Bluetooth. Finally, the IPG could work with the physician’s instructions (Figure 1A).

To introduce briefly, the remote programming system consists of three modules: the patient management module, teleprogram module, and video communication module. More details can be found in our patents (Chen et al., 2015a,b,d).

The patient management module was designed to build a stable connection between patients and physicians. A list of the patients and their status is shown on the webpage of the patient management module. The physician could only have access to his or her own patients. Once a patient logs into the system. They send their connection request to the physician. The physician could then choose to accept the request and establish a logical connection with the patient. At the same time, the management module would restrict the physician’s permission to connect with other patients.

The teleprogram module was designed to provide a platform for SCS remote programming. After a patient is connected to the physician, the patient listens to programming instructions from the server station, which were sent by the physician from the teleprogram module. A telemetry command will then be sent before the programming begins to ensure that all of the hardware or information links are in place. The physician’s computer client could store the patient’s medical records, electrode images, and previous programming records (Figure 1B). Healthcare providers can easily implement the adjustments of stimulation parameters (including contact mapping, selection of frequency, amplitude, pulse width, etc.), battery status check, electrode impedance check, and device troubleshooting on the teleprogram module. The programming records of every session will be stored in the physician’s client, uploaded to the database, and sent back to patients.

A video-recording function was developed based on the Web Real-Time Communication (WebRTC) technology. An open-source project of the STUN protocol was built to support the main signaling service, including taking advantage of the TURN protocol to penetrate firewalls if direct P2P connectivity cannot be established.

Furthermore, the patient’s client was designed as a smartphone terminal aimed at assisting patients in interaction with physicians, symptom management, and setting new adjustment parameters. With this client, patient could choose a physician and make a reservation for the remote programming service at home. Medical history, daily outcome reports, and uploaded imaging examinations of the patients are helpful in the management of symptoms and communication with physicians. As for the patients, the only potential restriction is an unstable network. The smartphone app will initially test the network and pair the programmer, and it then alerts the patients if an unstable connection occurs or pairing fails.

### Technological Considerations

The top two concerns of the remote programming are safety and confidentiality. Several appropriate data transmission protection technologies were adopted to ensure the security and confidentiality of communication, such as Hypertext Transfer Protocol Secure (HTTPS) between server and clients, bidirectional identity authentication technology based on digital certificate and short-distance wireless transmission with Bluetooth data transmission technology. The telemedicine system integrates multi-level data isolation and validation procedures without losing real-time performance. Transmission of programming instructions is encrypted between the mutual identity authenticated servers and clients. Also, safety protection mechanisms include functions of automatic instruction back-up, offline parameter recovery, and emergency stimulator shutdown. All of them are available and can be controlled by either programmer or patient. Furthermore, the notes from each encounter can be uploaded to a cloud-based database and can be accessed by any practitioner from any specialty at any time.

However, accidental disconnection resulted from unstable network, or inappropriate amplitude selection may cause severe adverse event. Unlike face-to-face interaction in hospital in which physicians could solve the problem immediately, we suggest at least one caregiver could keep accompany with the patient during the programming process. All patients and at least one caregiver should be made familiar with the remote programming and be able to cooperate with investigators to complete the whole procedure before leaving the hospital. Also, an incremental increase of the amplitude is recommended. In case any discomfort of patient or disconnection happens, the caregiver could restore the pre-programming settings with “one key recovery” or even shutdown the stimulator as quickly as possible.

## Results

The system was launched in 2019, and over 20 patients received this remote-programming-equipped G122R SCS (PINS Medical, Beijing) implantation at our center. From January 23, 2020, the date of lockdown of Wuhan, China, to April 30, 2020, 34 sessions for 16 patients with remote programming were implemented (Table 1). All of the programming practices were performed by a single doctor (Yang Lu) at his home or from our hospital in Beijing. The 16 patients underwent a total of 34 remote programming sessions, with an average adjustment frequency of 2.1 times/patient; the average program control time/adjustment was 43.1 ± 15 min (13–90 min) according to the records of the database.

**TABLE 1:** Characteristics of the 16 patients who received remote programming during the COVID-19 pandemic.

No.	Gender	Age	Diagnosis	Operation date	Programming times	Reason	Outcome	Patient’s rating for the remote programming
1	M	62	Periphery nerve injury	2019/11/5	3	Therapeutic effect decrease	Parameters adjustment (little improvement)	User-friendly
2	M	62	CRPS	2019/7/11	5	Therapeutic effect decrease (abnormal impedance of contacts)	Parameters and configuration adjustment (considerate improvement)	Connection unstable (resolved with changed network)
3	F	54	FBSS	2019/12/25	2	Therapeutic effect decrease	Parameters and configuration adjustment (considerate improvement)	User-friendly
4	F	55	Neuropathic pain	2019/12/24	2	Therapeutic effect decrease	Parameters and configuration adjustment (considerate improvement)	User-friendly
5	M	56	Amputated limb pain	2019/12/31	3	Therapeutic effect unstable	Change to sub-threshold stimulation (considerate improvement)	User-friendly
6	M	74	FBSS	2019/8/13	2	Intolerance to the traditional stimulation pattern	Oral comforting with lower amplitude	Connection unstable
7	M	55	Brachial plexus avulsion	2019/5/23	1	Therapeutic effect decrease	Parameters adjustment (no improvement)	Connection unstable (resolved with changed network)
8	M	55	Phantom limb pain	2020/1/3	3	Therapeutic effect decrease	Parameters and configuration adjustment (considerate improvement)	Connection unstable (resolved with changed network)
9	F	70	Spinal cord injury	2019/7/17	3	Therapeutic effect unstable	Parameters and configuration adjustment (some improvement)	User-friendly
10	M	74	Spinal cord injury	2019/7/4	1	Therapeutic effect decrease	Parameters adjustment (little improvement)	Connection unstable
11	F	73	FCSS	2019/10/28	2	Therapeutic effect unstable	Parameters and configuration adjustment (some improvement)	User-friendly
12	F	39	Spinal cord injury	2019/8/13	1	Red incision, without fever or pain	Continuous follow-up	User-friendly
13	M	68	Syringomyelia	2019/7/29	2	Therapeutic effect decrease	Parameters and configuration adjustment (some improvement)	User-friendly
14	M	48	Lower limb ischemic pain	2019/8/6	1	Therapeutic effect decrease	Parameters and configuration adjustment (some improvement)	User-friendly y
15	F	57	Brachial plexus avulsion	2019/7/17	1	Intolerance to the traditional stimulation pattern	Parameters adjustment (little improvement)	User-friendly
16	M	59	Periphery nerve injury	2019/12/9	2	Therapeutic effect decrease	Parameters adjustment (little improvement)	User-friendly

*CRPS, complex regional pain syndrome; FBSS, failed back surgery syndrome; FCSS, failed cervical surgery syndrome.*

Our cohort has different types of SCS indications. In contrast to the most common indication for SCS in United States is failed back surgery syndrome (FBSS), spinal-cord-injury-related pain is the most common indication in China. This suggests that patients that undergone more remote programming episodes likely underwent an SCS operation more recently. However, one patient with complex regional pain syndrome completed the remote programming five times owing to the poor quality of the internet connection and device failure (abnormal contact impedance).

Given the thorough penetration of smartphones in China, most of the patients could complete the service on their own or with the assistance of caregivers. Even for the four patients over the age of 70 years who do not use smartphones often, caregivers could help them to proceed with the entire remote programming session.

Thirteen of the 16 patients required programming for parameter optimization. This improvement was achieved with programming adjustment in 12 of 13 cases. Two of the 16 patients sought help because of intolerance to the induced paresthesia with a high amplitude. This is a typical dilemma for the patients using traditional low-frequency stimulation, which requires achieving a balance between the therapeutic effect and paresthesia. The last of the 16 patients experienced reddening of the skin near the incision site but no pain or fever. Since there were no clinical signs of infection, a subsequent follow-up was suggested for monitoring.

Eleven of the 16 patients reported that the system was user-friendly and met their needs. Five patients complained of an unstable connection resulting from a low network speed, three of whom solved this problem by changing the hotspot or using a 4G network. The other two patients could not solve this problem owing to limited resources. However, these two patients were able to complete the parameter adjustment with our adamant efforts and many reconnection attempts (over 2 h per patient). No adverse events occurred in the 34 programming sessions.

## Discussion

With regard to neuromodulation therapies, such as SCS, deep brain stimulation (DBS), vagal nerve stimulation, and sacral nerve neuromodulation, implantation of the device is only the first step, and post-operative programming is important for achieving long-term curative effect. Previous studies have proposed that remote programming technology could provide a safe, reliable, and efficient programming service for patients with reduced time and financial cost (Zhang et al., 2019, 2020b). This study represents the first introduction of remote programming technology for patients with chronic pain implanted with an SCS device.

Similar to DBS, patients implanted with an SCS device could receive programming in a face-to-face fashion or adjust the parameters by themselves at home within a limited range preset by the physicians. Self-adjustment has proved to be feasible and practical, and it significantly reduces the consultation time (Bally et al., 2019). However, this self-adjustment also needs to be performed under the supervision of the treating physicians, and patients cannot deal with a specific contact change or hardware malfunction by themselves. This had led to a huge demand for a remote programming system for these neuromodulation therapies, especially under extreme conditions such as the current COVID-19 pandemic.

For the patients with unsatisfactory post-operative outcomes with traditional parameters or configurations, many novel technologies have been developed in the field of neuromodulation therapies over the last few years, including current-based programming, interleaved programming, fractionated current, variable frequency stimulation, and directional current-steering technologies in DBS (Jia et al., 2017; Wagle Shukla et al., 2017) as well as 10-kHz high-frequency stimulation (Kapural et al., 2015), burst stimulation (Ahmed et al., 2018), and high-density stimulation in SCS (De Jaeger et al., 2017). These new paradigms require a deep understanding of the underlying therapies. Some stimulation patterns require several hours to several days to achieve a real and optimal response. There is no doubt that a physician’s operation and follow-up are inevitable additions to these patterns. Remote programming enables physicians to switch between different stimulation patterns and observe the therapeutic effect efficiently and conveniently.

Several retrospective studies have explored the effect of the DBS remote programming (Zhang et al., 2020a,b), which showed that over 80% of the patients were satisfied with the remote programming. Evaluation of the patients’ symptoms is always a challenge for DBS remote programming. Bradykinesia, tremor, and dyskinesia are easy to follow over video, whereas rigidity is more of a challenge to monitor remotely. In addition, it usually takes longer for the therapeutic effects of DBS to be evident for symptoms such as dyskinesia and tremors. By contrast, patients with pain could receive a real-time response to the stimulation in most cases. In this regard, symptom evaluation is much easier for the SCS remote programming relative to DBS.

Furthemore, pain treatment is not only about pain suppression itself, which has been particularly evident during the COVID-19 pandemic (Cohen et al., 2020) and has also emerged in this study. Within the 34 programming sessions, many patients expressed their anxiety and stress about their situation, especially those who were receiving inadequate care owing to greater isolation due to the lockdown. Anxiety and hopelessness are exacerbated by social isolation, and these negative moods aggravate the pain in a vicious cyclical manner. Thus, it is important to deliver palliative care for these patients in a timely manner. Healthcare providers need to be aware that many of the behavioral components of remote programming are not only potentially helpful for managing pain but also for emotional distress related to the COVID-19 pandemic. A retrospective study on DBS remote programming also found that some patients were satisfied with the programming sessions with no adjustment of the parameters required during the COVID-19 pandemic. This could be due to their expectations and the quality of the psychological support they received during the programming sessions (Zhang et al., 2020a). Empowered by the real-time video-based remote programming system, the demand for management of emotional distress could be met. We believe that reassuring SCS patients that they will be supported and their problems will be addressed during this pandemic at home is as important as the remote programming process.

Remote programming is cost-effective and convenient for patients with SCS and their families. However, before the interaction with healthcare providers can be efficiently established, several preparations need to be in place; these may include measures that ensure patients or their families know to use a smartphone and to keep updating the programming application using a stable network environment. As patients and at least one caregiver are requested to be familiar with the remote programming system, we expect to continue to face obstacles such as communication failure, poor network speed, and difficulty in following the orders when using the smartphone on their first attempt. In addition, a remote programming session is usually more laborious and time-consuming than face-to-face programming. For healthcare providers, patience and practice will help to ensure effective and efficient implementation of remote programming. For technology companies, improving the quality and efficiency is always a goal. We believe that with the continued development of technology, the system could become more intelligent, efficient, and effective at low cost.

If an increasing number of patients access to the remote programming service, the workflow should be designed to minimize the healthcare provider’s burden. The teleprogramming team should be organized, including physicians, nurses, and technicians. Team cooperation could schedule visits, remotely train patients and families, resolve technological problems, and optimize the workflow. We suggest that before the start of formal remote programming, team members should recheck the schedule, smooth the network, and ensure patient and family computer literacy.

At the beginning of 2020, the whole world witnessed this unprecedented pandemic; everyone is a fighter against the invisible enemy, and we must all practice “social distancing.” During this invisible war, patients with chronic intractable pain with an implanted SCS implant suffer from exacerbated pain helplessly and cannot receive individualized programming on site under these circumstances of isolation. We have demonstrated that the PINS remote wireless programming system allows for connection between patients and healthcare providers within this context, delivers safe and effective remote programming services, and provides palliative care value to the most vulnerable population of chronic pain patients.

With clinical implementation and feedback, we concluded that this remote wireless programming system is an effective and safe method for delivering parameter adjustment, thus improving the quality of life of chronic pain patients.

## Data Availability Statement

The raw data supporting the conclusions of this article will be made available by the authors, without undue reservation.

## Ethics Statement

The studies involving human participants were reviewed and approved by Ethics committee of Beijing Tsinghua Changgung Hospital. The patients/participants provided their written informed consent to participate in this study. Written informed consent was obtained from the individual(s) for the publication of any potentially identifiable images or data included in this article.

## Author Contributions

GW and LL contributed to the conception, the study design, the acquisition, the analysis, the interpretation of data, and the preparation of the manuscript. JW contributed to the study design and the editing of the manuscript. YL contributed to the acquisition of data, manuscript writing, and the editing of the manuscript. DX contributed to the study design, the interpretation of data, and editing of the manuscript. SD and XZ contributed to the study design, the analysis, and the interpretation of data. BY and HZ contributed to the interpretation of data and the editing of the manuscript. All authors contributed to the article and approved the submitted version.

## Conflict of Interest

JW and GW have received research support from Beijing PINS Medical Co., (donated SCS devices for pain). LL reports personal fees from Beijing Pins Medical Co., outside the submitted work. The remaining authors declare that the research was conducted in the absence of any commercial or financial relationships that could be construed as a potential conflict of interest.
